# Satellite cells delivered in their niche efficiently generate functional myotubes in three-dimensional cell culture

**DOI:** 10.1371/journal.pone.0202574

**Published:** 2018-09-17

**Authors:** Johanna Prüller, Ingra Mannhardt, Thomas Eschenhagen, Peter S. Zammit, Nicolas Figeac

**Affiliations:** 1 King's College London, Randall Centre for Cell and Molecular Biophysics, New Hunt's House, Guy's Campus, London, England; 2 Department of Experimental Pharmacology and Toxicology, University Medical Center Hamburg Eppendorf, Hamburg, Germany; 3 DZHK (German Centre for Cardiovascular Research), Hamburg, Germany; University of Minnesota Medical Center, UNITED STATES

## Abstract

Biophysical/biochemical cues from the environment contribute to regulation of the regenerative capacity of resident skeletal muscle stem cells called satellites cells. This can be observed *in vitro*, where muscle cell behaviour is influenced by the particular culture substrates and whether culture is performed in a 2D or 3D environment, with changes including morphology, nuclear shape and cytoskeletal organization. To create a 3D skeletal muscle model we compared collagen I, Fibrin or PEG-Fibrinogen with different sources of murine and human myogenic cells. To generate tension in the 3D scaffold, biomaterials were polymerised between two flexible silicone posts to mimic tendons. This 3D culture system has multiple advantages including being simple, fast to set up and inexpensive, so providing an accessible tool to investigate myogenesis in a 3D environment. Immortalised human and murine myoblast lines, and primary murine satellite cells showed varying degrees of myogenic differentiation when cultured in these biomaterials, with C2 myoblasts in particular forming large multinucleated myotubes in collagen I or Fibrin. However, murine satellite cells retained in their niche on a muscle fibre and embedded in 3D collagen I or Fibrin gels generated aligned, multinucleated and contractile myotubes.

## Introduction

Skeletal muscle represents the most abundant tissue in man, comprising approximately 38% of total body weight in males, and 30% in females [1]. Skeletal muscle contraction controls voluntary body movement, from postural maintenance to locomotion, as well as having important metabolic functions and generating heat for body temperature regulation.

Skeletal muscles are composed of multiple muscle fibres. These large, cylindrical, multinucleated and contractile cells are formed from the fusion of many myoblasts. Contractile function of muscle fibres is controlled by innervation, and supported by a network of blood vessels and connective tissue and extracellular matrix (ECM). Amongst the ECM proteins characteristically found in skeletal muscle are collagens (e.g. collagens I, III and IV), proteoglycans (syndecans, decorin), fibronectin and laminin [2].

Adult myofibres are post mitotic but skeletal muscle has a remarkable capacity to repair/regenerate after certain types of damage due to resident muscle stem cells called satellite cells [3–5]. Satellite cells are mitotically quiescent in adult skeletal muscle and reside beneath the basal lamina surrounding each muscle fibre. After damage, satellite cells are activated to proliferate to provide myoblast progeny, that subsequently differentiate to form nascent muscle fibres, or repair damaged myofibres by supplying new myonuclei by fusing with them. Satellite cells also self-renew to maintain a viable stem cell compartment [6].

In large volume muscle injuries or some chronic diseases such as muscular dystrophies however, damage may exceed the capacity for functional regeneration [7]. In volumetric muscle loss, the reparative, stabilizing fibrotic response inhibits new muscle formation. Dense fibrotic tissue can also inhibit neural and vascular ingrowth to render muscle denervated and ischaemic. In chronic degenerative diseases such as Duchenne muscular dystrophy (DMD), muscle continuously cycles between degeneration and regeneration, which eventually result in fibrosis, fat accumulation, and a decline in regenerative potential of satellite cells [8]. With aging, loss of muscle mass and function is amplified by decrease in satellite cell numbers and an impairment of their regenerative capacity due to intrinsic and extrinsic factors [9].

Modelling muscle formation/regeneration *in vitro* is essential to both understand the process, and how to generate sufficient satellite cells/muscle for therapeutic grafting. *In vitro* expansion of satellite cells though, can quickly cause loss of their regenerative potential [6, 8, 10–12]. In addition to various small molecules that can increase satellite cell expansion ex-vivo [13, 14], properties of the culture substrate is also a factor [10]. This is unsurprising, since components of the ECM are essential to support the regeneration process [15]. For example, collagen V and VI in the satellite cell niche is essential to prevent exhaustion of the satellite cell pool [16, 17] and laminin in the niche is actively remodelled during repair [18]. Furthermore, factors in the niche also support muscle regeneration by stimulating growth of blood vessels (VEGF, angiopoietin) or innervation of the newly formed muscle fibres (NERFs) [19, 20]. Thus, efficient muscle fibre formation and restoration of the ECM, vascularisation and innervation must be coordinated for effective and functional muscle regeneration.

Strategies to enhance muscle repair *in vivo* can be broadly divided into three (overlapping) groups: (i) administration of factors to enhance myogenesis: such as IGF, VEGF, NO, Wnt7a [21–25]; (ii) administration of cell types known to contribute to muscle regeneration: including satellite cells, pericytes and CD133+ cells [26–31]; (iii) grafting of *in vitro* engineered muscle tissue [32, 33]. Cells and factors are often encapsulated in 3D scaffolds for delivery, not only because the scaffold biomaterial can enhance the regenerative process, but it can also be used to control issues such as the dynamics of substance/cell release [26]. However, only engineering large skeletal muscle tissue *in vitro* would be a suitable approach to treat volumetric muscle loss, when the endogenous repair abilities are insufficient.

Scaffolds for skeletal muscle engineering should (i) be biocompatible, (ii) support myoblast proliferation and differentiation, (iii) allow vascularisation and innervation and (iv) facilitate directional alignment for optimal force production, (v) be remodelled/degraded. Common scaffold biomaterials used in muscle engineering are collagen, hyaluronan, fibrin, alginate and polyethylene glycol [26]. In this study, we focus on collagen type I and fibrin, as well as a PEG-fibrinogen blend, since these biomaterials are proven to support myogenesis.

Collagen is a major structural protein in skeletal muscle ECM, accounting for 1–10% of dry muscle mass weight, and has been shown to support proliferation, differentiation and myotube formation of immortalised and primary murine myoblasts [34–42]. Fibrin is a biopolymer commonly used in tissue engineering [43]. It polymerises after thrombin mediated cleavage of fibrinopeptides A and B, and modulation of thrombin amount allows tuning of physical properties such as stiffness, pore and branch size [44, 45]. Fibrin contains an abundance of cell attachment sites that interact with integrins and growth factors (e.g. VEGF, bFGF2) [46–48], it is biocompatible and degradable, and supports and improves myogenesis. Indeed, culture of Human Muscle Derived Cells (MDCs) in Fibrin gel produces an engineered skeletal muscle with structural resemblance to *in vivo* tissue [49]. Complementary, co-culture of rat primary embryonic motor neurons and neonatal myoblasts in three-dimensional Fibrin hydrogels allows formation of functional neuromuscular junctions [50]. Also using myogenic neonatal rat cells, a highly functional biometric muscle tissue has been engineered using Fibrin, complete with a populated satellite cell niche, the ability for vascular integration, and functional *in vivo* maturation [51]. Finally, PEG based hydrogels are also cytocompatible and tuneable in regard to mechanical properties [52] or adhesion sites. Photopolymerizable PEG-fibrinogen hydrogel is clinically approved and generates a favourable microenvironment by coupling natural and synthetic features [53]. PEG-fibrinogen supports myogenic differentiation, cell survival after transplantation and angiogenic infiltration *in vitro* and *in vivo* [54, 55]. Murine mesoangioblasts overexpressing placenta derived growth factor (PDGF) encapsulated in a PEG-fibrinogen scaffold generated a functional artificial muscle [32].

Maturation of a construct can be achieved by non-directional mechanical or electrical stimulation [56–59], but alignment is achieved when directional force is applied to the cell containing scaffold, either actively or passively [34, 60, 61]. In this study, we employed a model originally used for engineered heart tissue (EHT) [60]. Polymerisation of the biomaterial between two anchor points, mimicking tendon anchorage, allows exertion of stress on the encapsulated cells, which is sufficient to promote alignment along the principle axis of force. This 3D culture system is simple, fast to setup, inexpensive and reproducible, making it a useful tool to screen myoblast behaviour in different 3-dimensional biomaterials.

This study aimed to examine in parallel the ability of collagen I, Fibrin and PEG-Fibrinogen in an *in vitro* 3D environment to support myogenesis from multiple myoblasts types including immortalized murine (C2C12) [62] and human myoblasts (C25Cl48) [63], primary expanded murine satellites cells [64], and satellite cells retained in their niche on a freshly isolated muscle fibre [65].

## Materials and methods

### Biomaterials

#### Rat collagen I

80% of type-I acid soluble collagen (First link, UK: rat tail collagen1: 2.05 mg/ml, cat:60-30-810) was mixed on ice with 10% of 10x MEM (Gibco, UK). Collagen solution was neutralised with 1 M NaOH until colour change from yellow to bright pink. Neutralised solution was left on ice for 30 minutes, mixed with cells or muscle fibres and incubated at 37°C for 30–60 mins to allow polymerisation (Table 1). Polymerised Collagen I at a concentration of 1.5 mg/ml has a stiffness of ~0.2 KPa [66].

**Table 1:** Biomaterials.

Biomaterial	Source	Final concentration	Gel volume	Polymerisation
Rat collagen I	First link: cat: 60-30-810	±1.5 mg/ml	100 μl	Between, 2 silicone posts, 30–60 min at 37°C
Bovine fibrinogen, Thrombin, Aprotinin (in medium)	Sigma: F8630, Baxter: Tissuco Duo 500, Sigma: A1153	5 mg/ml, 3 U/ml, 33 μg/ml	100 μl	Between, 2 silicone posts, 30–60 min at 37°C
Bovine fibrinogen, Thrombin, Aprotinin (in medium), Plus Matrigel (Growth Factor Reduced Basement Membrane Matrix)	Sigma: F8630, Baxter: Tissuco Duo 500, Sigma: A1153, (BD: 354230)	5 mg/ml, 3 U/ml, 33 μg/ml, 1mg/ml	100 μl	Between, 2 silicone posts, 30–60 min at 37°C
PEG-Fibrinogen, Irgacure	TECHNION, Sigma: 410896	8 mg/ml, 0.1%	100 μl	In 96 well plate under UV (365nm) for 10–20 min

#### Bovine fibrinogen

A stock solution of bovine fibrinogen (Sigma: F8630) was prepared in sterile PBS (100 mg/ml) and stored at -20°C. The stock solution was diluted to 5 mg/ml in proliferation medium. Fibrinogen was then mixed with cells or muscle fibres and Thrombin (Tissuco Duo 500, Baxter, Illinois, USA) was added on ice at a final concentration of 3 U/ml. The solution was quickly mixed and incubated at 37°C for 30–60 mins to polymerise. To avoid Fibrin degradation, Aprotinin (Sigma A1153) was added at a final concentration of 33 μg/ml in culture medium (Table 1). Polymerised Fibrin gel formed using 5 mg/ml of Fibrinogen and 3U/ml of Thrombin has a stiffness of ~1±0.1 KPa [67] [68]. As a positive control for some experiments, 10% (volume) of Matrigel (Growth Factor Reduced Basement Membrane Matrix) (BD 354230) was added to the Fibrin gel to a final concentration of ~1 mg/ml.

#### Polyethylene glycol Fibrinogen (PEG-Fibrinogen)

PEG-Fibrinogen (8–10 mg/ml) was provided by Technion and stored at -80°C. Once defrosted, 0.1% of Irgacure (Sigma: 410896) (stock solution: 10% of Irgacure diluted in ethanol 70%) was added to the PEG-Fibrinogen. Cells or muscle fibres were mixed with the solution, transferred into a 96 well plate and cross-linked under an UV light (365 nm) (3UV Lamp, 230 V, 50 Hz, ThermoFisher) for 10 mins. Once polymerised, the gel was transferred to a 24 well plate, incubated in proliferation medium for several days and then switched to differentiation medium to induce myotube formation (Table 1). Polymerised PEG-Fibrinogen gel constituted with 7 mg/ml has a stiffness of ~0.125 KPa [69]. Cytotoxicity of UV light (365 nm) used to initiate PEG-FN polymerisation was previously tested and found to be minimal [70] and the same parameters were used here [32, 54].

### 3D culture model

**Fig 1:**
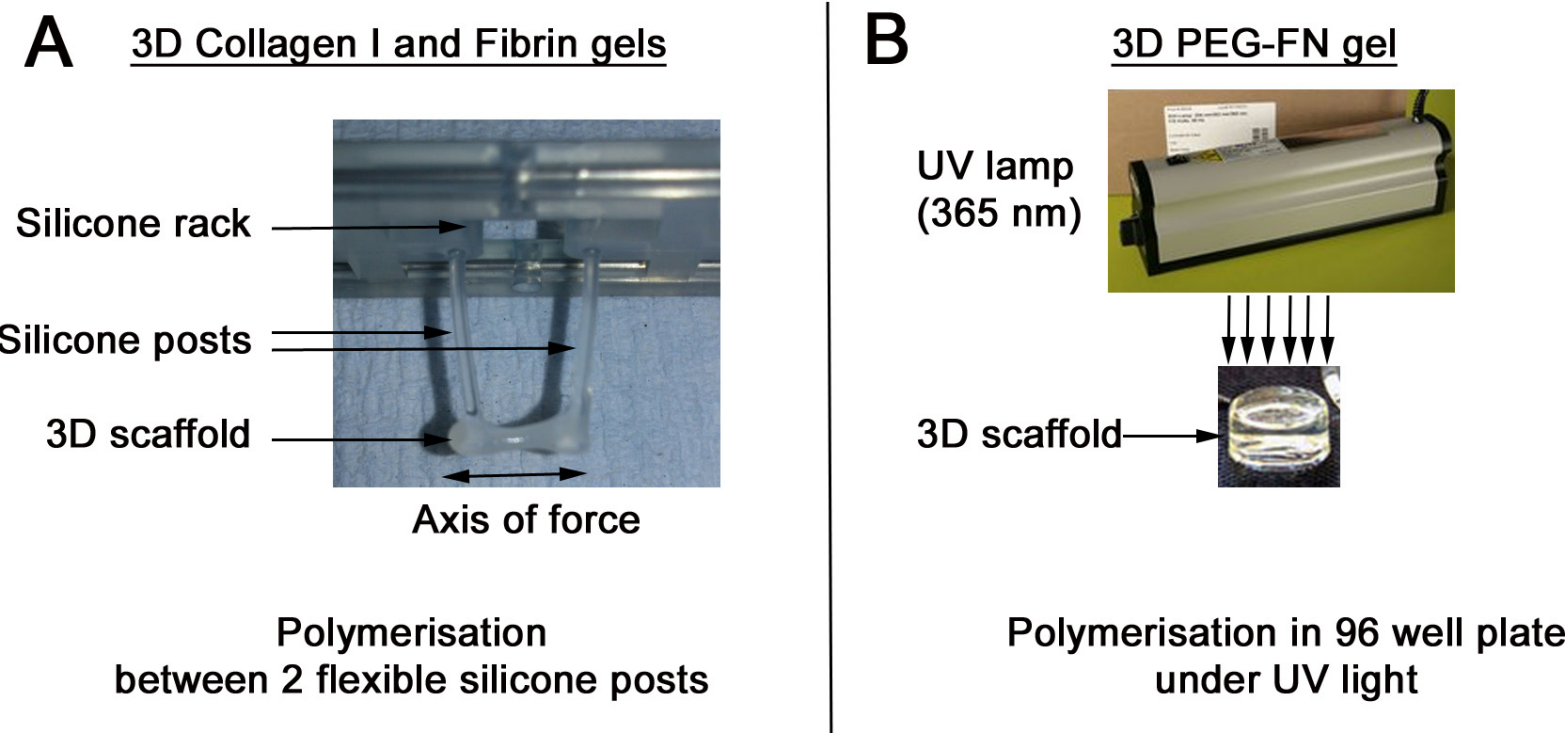
Experimental setup to generate 3D gels. Collagen and Fibrin gels were polymerized between two silicone posts that mimic tendons (A). The biomaterial was mixed with muscle cells or muscle fibres. A pair of flexible silicone posts supported by a silicone rack was immersed in the gel. The gel containing the cells polymerises between the two silicone posts. After polymerisation, the silicone rack holding the silicone posts was used to transfer the gel into a 24-well tissue culture dish. The axis of force created by the silicone posts promotes cellular alignment. PEG-Fibrinogen could not to polymerised between the two silicone posts and was instead polymerised in a 96 well plate using UV light (B). Once polymerised, the gel was transferred to a 24 well plate, but undergoes no uniform, directed static strain.

This method was adapted from that used for cardiac muscle [60]. Collagen and Fibrin gels were polymerized between 2 silicon posts that mimic tendons (Fig 1A) [60]. The biomaterial (100 μl/gel) was mixed with muscle cells (around 0.5 million cells/gel) or with muscle fibres (100 fibres/gel). The cell/biomaterial combination was then cast in a rectangular agarose-casting mold (12 x 3 x 3 mm) prepared in a standard 24-well cell culture dish. A pair of flexible silicone posts supported by a silicone rack was immersed in the gel. After polymerization (30–60 mins at 37°C), a 3D gel containing the cells is formed between the two silicone posts. When polymerised, the silicone rack holding the silicone posts is used to transfer the gel into a 24-well tissue culture dish containing proliferation medium. In culture, cells remodel the gel and create tension that promotes alignment along the principal axis of force generated between the posts. To synchronise initiation of myogenic differentiation, proliferation medium was changed to differentiation medium.

PEG-Fibrinogen did not polymerase between the two silicone posts and was instead polymerised in 96 well plates (Fig 1B). Once polymerised, the gel was transferred to a larger plate (24 well plates). In this context, no static strain is applied on this scaffold to promote myotubes alignment. The size of the gel after polymerisation was about 8.5 mm in length and 1–2 mm in diameter.

### Myoblast sources

#### Murine C2C12 myoblasts

Immortalized murine C2C12 myoblasts [62] were cultured in proliferation medium (DMEM supplemented with L-glutamine (2 mM), 10% foetal bovine serum (FBS) and 1% penicillin–streptomycin. For differentiation, myoblasts were switched to low-mitogen differentiation medium (DMEM supplemented with 2 mM L-glutamine, 2% Horse Serum (HS) and 1% penicillin–streptomycin) (Table 2).

**Table 2:** Muscle cell sources and cell culture conditions.

Muscle cells type	Number of cells per gel	Proliferation medium	Differentiation medium
Murine immortalized C2C12 myoblasts	450 000 myoblasts	DMEM supplemented with L-glutamine (2mM), 10% foetal bovine serum (FBS) and 1% penicillin–streptomycin, 2 days	DMEM supplemented with L-glutamine (2mM), 2% HS and 1% penicillin–streptomycin, 3 days
Immortalized human myoblasts C25Cl48	500 000 myoblasts	Promocell, Skeletal Muscle Cell Growth Medium Kit (C-23160) supplemented with 20% FBS and Gentamycin (50 μg/ml), 2 days	Promocell, Skeletal Muscle Cell Differentiation, Medium Kit (C-23161) supplemented with gentamicin (50μg/ml), 6 days
Primary *in vitro* expanded satellite cell-derived myoblasts from EDL muscle	500 000 myoblasts	DMEM-GlutaMAX, 30% FBS, 10% HS, 1% CEE, 10 ng/ml bFGF, and 1% penicillin–streptomycin, 4 days	DMEM-GlutaMAX supplemented with 2% FBS and 1% penicillin–streptomycin, 2 to 10 days
Freshly isolated murine Soleus myofibres	100 myofibres	DMEM-GlutaMAX, 30% FBS, 10% HS, 1% CEE, 10 ng/ml bFGF, and 1% penicillin–streptomycin, 10 days	DMEM-GlutaMAX supplemented with 2% FBS and 1% penicillin–streptomycin, 3 days

#### Immortalized human myoblasts

Immortalized human myoblasts C25Cl48 [63] were maintained in cell growth medium (Promocell: C-23160) supplemented with 20% FBS and Gentamycin (50 μg/ml). For differentiation, C25Cl48 were switched to skeletal muscle cell differentiation medium (C-23161: Promocell) with gentamicin (50 μg/ml) (Table 2).

#### Primary murine expanded satellite cells

Adult C57BL10 mice (8–12 weeks old) were killed by cervical dislocation and the *Extensor digitorum longus* muscle (EDL) was removed and digested in 0.2% collagenase type 1 (sigma) for 2 h at 37°C [71]. Satellite cell-derived myoblasts were allowed to migrate from isolated myofibres cultured in proliferation medium (DMEM-GlutaMAX, 30% FBS, 10% HS, 1% chick embryo extract (CEE), 10 ng/ml bFGF, and 1% penicillin–streptomycin) on Matrigel. Three days later, myofibres were removed, satellite cells re-plated on Matrigel and expanded in proliferation medium for another 3 days before being embedded in a 3D scaffold. Satellite cells were then switched to differentiation medium (DMEM-GlutaMAX supplemented with 2% HS and 1% penicillin–streptomycin) (Table 2).

#### Satellite cells associated with a myofibre

Adult C57BL10 mice (8–12 weeks old) or *3F*-*nlacZ*-*E* mice [72] [73]. were killed by cervical dislocation and the Soleus muscle isolated and digested in 0.2% collagenase type 1 (sigma) for 120–135 mins at 37°C [65]. Freshly isolated myofibres were embedded in 3D scaffolds and cultured in proliferation medium (DMEM-GlutaMAX, 30% FBS, 10% HS, 1% CEE, 10 ng/ml bFGF, and 1% penicillin–streptomycin). In proliferative conditions, satellites were activated, proliferated and colonised the 3D scaffold. When approximately confluent, cells were switched to differentiation medium (DMEM-GlutaMAX supplemented with 2% HS and 1% penicillin–streptomycin) (Table 2).

### Ethical statement

Procedures were carried out under the Animals (Scientific Procedures) Act 1986, and this study was approved by the King's College London Ethical Review Process committee.

### EdU incorporation and immunolabeling

EdU (10 μM, Invitrogen Life Technologies) was added at a concentration of 10 μM for 2 hours, and the gels then fixed. 3D constructs were fixed with 4% paraformaldehyde/PBS for 20 mins. EdU incorporation was revealed using a Click-iT EdU Imaging Kit (Invitrogen Life Technologies) as per manufacturer's instructions.

For immunolabeling, gels were permeabilised with 2.5% Triton X-100/PBS for 15 min at room temperature and blocked with 5% goat serum/5% swine serum in PBS for 60 min at room temperature. Primary antibodies used were monoclonal mouse anti-MyHC (MF20-c DSHB, 1/100), anti-Desmin (clone D33, DAKO, 1/100), anti-β-Tubulin (E7-c, DSHB, 1/100) and monoclonal rabbit anti-Ki67 (Ab16667, Abcam, 1/100). Primary antibodies were diluted in blocking solution and applied overnight at 4°C. Following multiple washes in PBS/0.05% Tween 20, gels were incubated with fluorochrome-conjugated secondary antibodies (Molecular Probes) used at 1/250 for 90 min at room temperature and then 4, 6-diamidino-2-phenylindole (DAPI: 300 nM) in PBS for 20 min. All the steps were performed under agitation (rotating mixer) and washes were performed in PBS/0.05% Tween 20. β-galactosidase activity from the *3F-nlacZ*-*E* transgene was revealed using X-gal solution for 60 min at 37°C.

### Image acquisition and measuring

Images were acquired on a Zeiss Axiovert 200 M microscope using a Zeiss AxioCam HRm and AxioVision software version 4.4. Images of muscle fibres were acquired using a Zeiss Axiovert 25 microscope. Images were adjusted globally for brightness and contrast. Dimensions of gels were determined form images taken at a standard magnification with reference to a graticule.

### Quantitative RT-PCR

Total RNA was extracted using the RNeasy Kit (Qiagen) and cDNA prepared with the QuantiTect Reverse Transcription Kit with genomic DNA wipeout (Qiagen). RT-qPCR was performed on an Mx3005PQPCR system (stratagene) with Brilliant II SYBR green reagents and ROX reference dye (Stratagene). Human RT-qPCR primers for *TBP* (F: 5’-CGGCTGTTTAACTTCGCTTC-3’ and R: 5’-CACACGCCAAGAAACAGTGA-3’), *MYH2*,*3*,*8* (F: 5’-AGCAGGAGGAGTACAAGAAG-3’ and R: 5’-CTTTGACCACCTTGGGCTTC-3’), *ACTN3* (F: 5’-CAGCACCTGGCTGAGAAGTT-3’ and R: 5’-AGCAAAGCCGAATCGTAGTC-3’), *TPM1* (F: 5’-GGTCCTTTCCGACAAGCTG-3’ and R: 5’-TGGCATGAGCCACTTTCTCT-3’), *CKM* (F: 5’-ACCTCAACCATGAAAACCTCA-3’ and R: 5’-GGCTGCTGAGCACGTAGTTA-3’). Mouse RT-qPCR primers for *Tbp* (F: 5’-ATCCCAAGCGATTTGCTG-3’ and R: 5’-CCTGTGCACACCATTTTTCC-3’) *Myh1* (F: 5’-GTCCAAAGCCAACAGTGAAG-3’ and R: 5’-CTTCTGTTTCCATTCTGCCA-3’), *Actn3* (F: 5’-TGAACCAGGAAAATGAGAAGC-3’ and R: 5’-GCGGATCCACTCCAACAG-3’), *Tpm1* (F: 5’-CCAAATTGGAGAAAAGCATTG-3’ and R: 5’-TGGAAGTCATATCGTTGAGAGC-3’), *Ckm* (F: 5’-GCATCAAGGGTTACACTCTGC-3’ and R: 5’-CCCGTCAGGCTGTTGAGA-3’). Gene expression was normalized to *TBP* and measured from three independent samples and significant differences assessed using a Student's t-test.

## Results

### Collagen I, Fibrin and PEG-Fibrinogen 3D scaffolds support myogenic progression in murine C2C12 myoblasts

**Fig 2:**
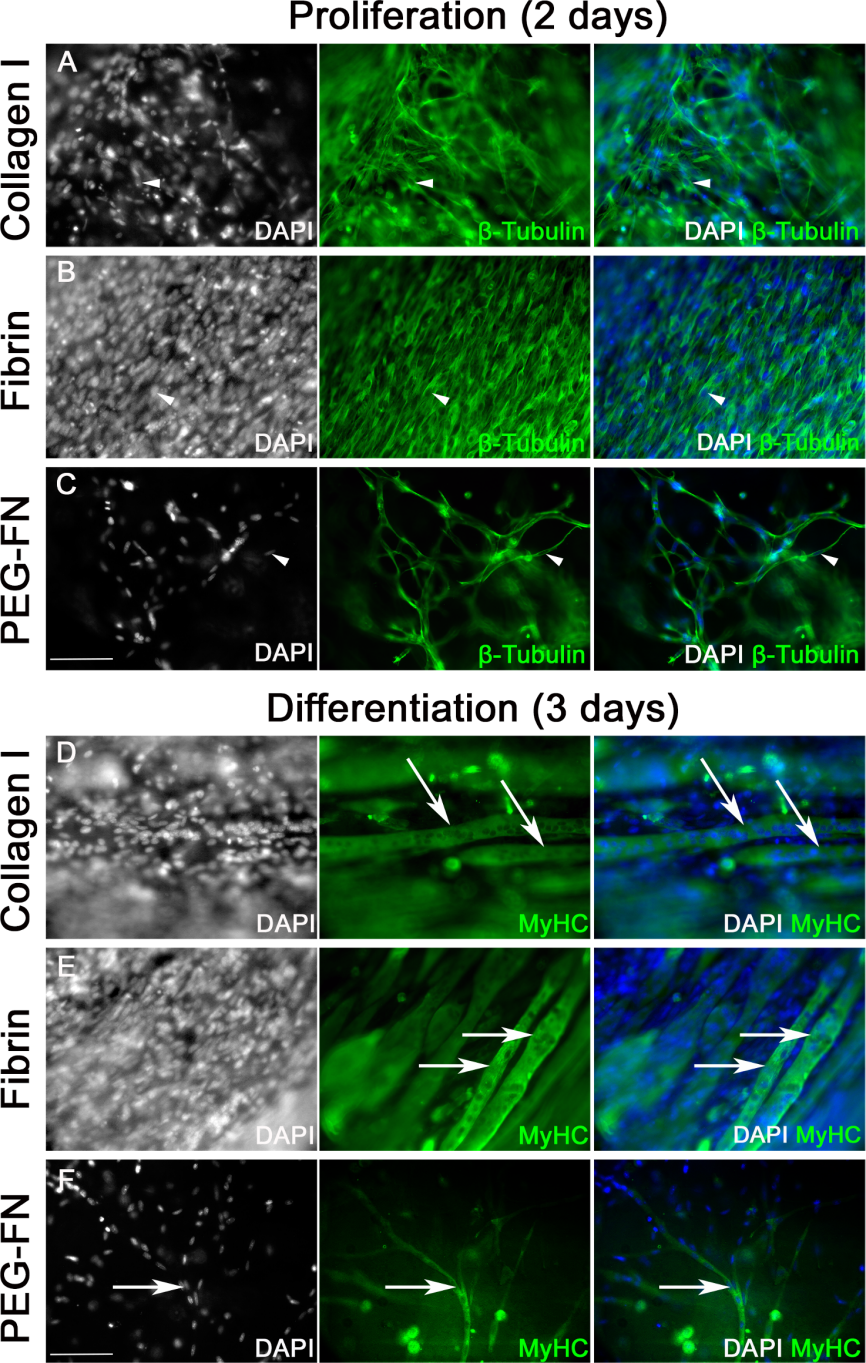
All 3D biomaterial scaffolds promote robust myotube formation of murine C2C12 myoblasts in vitro. Immortalized C2C12 myoblasts, embedded in Collagen I (450 000 cells / 100 μl: A and D) or Fibrin (450 000 cells / 100 μl: B and E) gels were polymerised between two silicone posts. PEG-Fibrinogen (300 000 cells / 100 μl: C and F) gels were polymerised in a 96 well plate and cultured without uniform static tension. Gels were cultured in proliferation medium for 2 days (A-C) and then switched to differentiation medium for 3 days (D-F). 3D scaffolds were fixed and immunolabelled for β-Tubulin (A-C) or myosin heavy chain (MyHC) (D-F) and nuclei counterstained with DAPI. In Collagen I (A) and Fibrin (B) gels, proliferating myoblasts were often aligned along the line of force created by the silicone posts and gel compaction was observed. In PEG-Fibrinogen gels (C), C2C12 myoblasts present randomly orientated membrane projections and no gel compaction was observed. After 3 days in differentiation medium, large parallel myotubes (arrowed) were observed in Collagen I (D) and Fibrin (E) gels. Thinner and randomly orientated myotubes were present in PEG-Fibrinogen scaffold (F). Scale bar represents 100 μm. Representative images from three or more independent experiments.

We first analysed myogenic progression of immortalized murine C2C12 myoblasts in collagen I, Fibrin and PEG-Fibrinogen 3D scaffolds. C2C12 have several advantages, including unlimited expansion and robust myotube formation in classic 2D cell culture, and are commonly used in muscle research. Immortalized C2C12 myoblasts were embedded in collagen I (Fig 2A and 2D), Fibrin (Fig 2B and 2E) or PEG-Fibrinogen (Fig 2C and 2F) gels. Collagen I and Fibrin 3D scaffolds were then polymerised between two flexible silicone posts to create tension that promotes myoblast alignment and myotube formation (Fig 1A). PEG-Fibrinogen could not be polymerased between the two silicone posts and was instead polymerised in 96 well plates (Fig 1B). Myoblasts were cultured in proliferation medium for 2 days (Fig 2A–2C) and then switched to differentiation medium for another 3 days (Fig 2D–2F).

After two days in proliferation medium, 3D scaffolds were fixed and immunolabelled for β-Tubulin to visualise the morphology of proliferating myoblasts (Fig 2A–2C). In collagen I (Fig 2A) and Fibrin (Fig 2B) gels, C2C12 myoblasts had an elongated shape and were generally aligned along the axis of force generated between the silicone posts. Alignment was more pronounced in Fibrin gels (Fig 2B), compared to collagen (Fig 2A). A compaction of collagen I and Fibrin gels was observed, since they are natural biomaterials that can be actively remodelled by cells. Indeed, human myoblasts actively stiffen 3D Fibrin gels [67]. In PEG-Fibrinogen gels, C2C12 myoblasts also showed an elongated morphology but were randomly orientated (Fig 2C). No obvious remodelling of the gel was observed.

After 3 days in differentiation medium, scaffolds were fixed and immunolabelled for myosin heavy chain (MyHC) to monitor sarcomeric assembly in myotubes (Fig 2D–2F). Large multinucleated myotubes with parallel alignment were observed in both collagen I (Fig 2D) and Fibrin (Fig 2E) gels. A higher density of large myotubes was present in Fibrin gels. Multinucleated myotubes were also present in PEG-Fibrinogen scaffold (Fig 2F) but were thinner and not aligned. Absence of static tension, combined with reduced gel compaction that effectively decreased cell density, could explain this observation. However, reduction of cell proliferation and myotube differentiation due to the biochemical and biophysical nature of the PEG-Fibrinogen can not be excluded.

These results show that all three biomaterials in a 3D culture support myogenic progression of C2C12 myoblasts, from cell attachment, elongation, proliferation, alignment to myogenic differentiation to fusion into multinucleated myotubes.

### Collagen I, Fibrin and PEG-Fibrinogen 3D scaffolds support moderate myogenic progression of immortalized human myoblasts

**Fig 3:**
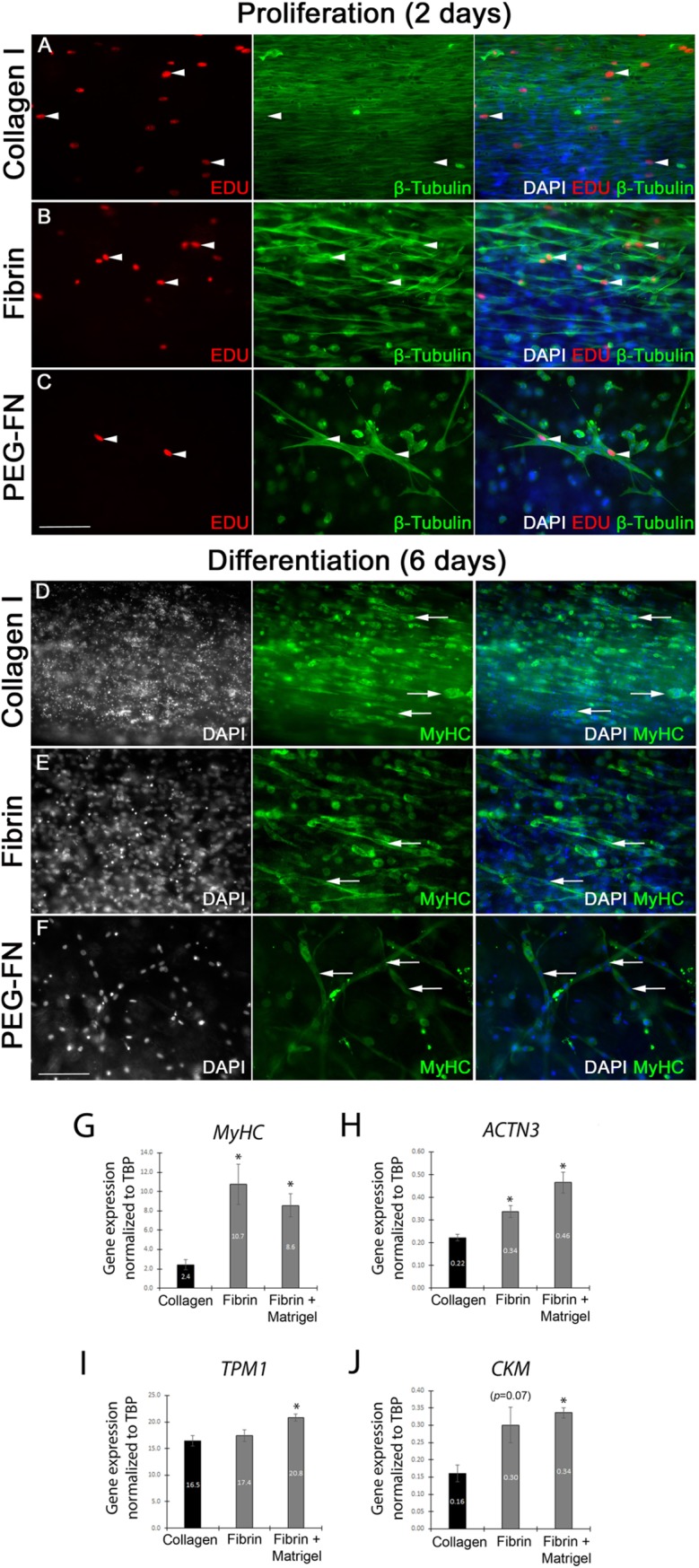
Myogenic progression of immortalized human myoblasts in different 3D biomaterial scaffolds in vitro. Immortalized human myoblasts C25Cl48 were embedded in collagen I (500 000 cells / 100 μl: A and D), Fibrin (500 000 cells / 100 μl: B and E) or PEG-Fibrinogen (500 000 cells / 100 μl: C and F), cultured in proliferation medium for 2 days (A-C) and then switched to differentiation medium for 6 days (D-F). After 2 days in proliferation medium, cells were pulsed with the thymidine analogue 5-Ethynyl-2’deoxyuridine (EdU) for 2 hours and then fixed, before EdU incorporation was visualised and myoblasts immunolabelled for β-Tubulin and nuclei counterstained with DAPI. After 6 days in differentiation medium, cells were fixed and immunolabelled for myosin heavy chain (MyHC) to visualise myotubes and counterstained with DAPI. Proliferating EdU positive (arrow-heads) and β-Tubulin myoblasts were aligned in collagen I (A) and Fibrin (B) gels but randomly orientated in PEG-Fibrinogen scaffold (C). After 6 days in differentiation medium many MyHC positive cells were present but only a few small myotubes were detected in collagen I (D) or Fibrin (E) gels. In PEG-Fibrinogen scaffold (C), small myotubes were randomly orientated. Scale bar represents 100 μm. Representative images from 3 or more independent experiments. Expression of the sarcomeric proteins *Myosin Heavy chain* (*MYH2*, *3*, *8*) (G), Actinin α3 *(ACTN3*) (H) and Tropomyosin 1 (*TPM1*) (I) and creatine kinase *(CKM*) (J) after 7 days of differentiation were analysed by RT-qPCR. Expression was normalized to the house keeping gene *TBP*. Data are mean±SEM from 3 independent gels where an asterisk denotes a significant difference (*p*<0.05) from the Collagen gel using an unpaired two-tailed Student's t-test.

We then tested the ability of the biomaterials to support myogenic progression of human myoblasts in a 3D environment, a model relevant for potential therapeutic application. Immortalized human C25Cl48 myoblasts were embedded in collagen I (Fig 3A and 3D), Fibrin (Fig 3B and 3E) or PEG-Fibrinogen (Fig 3C and 3F), cultured in proliferation medium for 2 days (Fig 3A–3C) and then switched to differentiation medium for 6 days (Fig 3D–3F). After two days in proliferation medium, cells were pulsed with the thymidine analogue 5-Ethynyl-2’deoxyuridine (EdU) for two hours and fixed, before being immunolabelled for β-Tubulin and EdU incorporation visualised using the Click-it Edu imaging kit.

In collagen I (Fig 3A) and Fibrin (Fig 3B) gels, myoblasts had an elongated morphology and were aligned along the axis of force created by the silicone posts. Human myoblasts were denser and more compact in collagen I compared to Fibrin, suggesting that human myoblasts interact differently with these biomaterials. Both collagen I and Fibrin scaffolds promoted myoblast proliferation, as many had incorporated EdU (Fig 3A and 3B). Human myoblasts elongated and proliferated in PEG-Fibrinogen, but appeared unable to remodel the biomaterial, as no gel compaction was observed, with fewer elongated human myoblasts present (Fig 3C).

After 6 days in differentiation medium, gels were fixed and immunolabelled for MyHC to assess myotube formation (Fig 3D–3F). While many MyHC positive cells were present, only a few small myotubes were evident, showing the ability of all three biomaterials to support myoblast differentiation but limited fusion. Myocytes/myotubes were aligned in collagen I (Fig 3D) and Fibrin (Fig 3E), but not in PEG-Fibrinogen (Fig 3F) scaffolds. In Collagen gels however, DAPI staining revealed many necrotic/apoptotic nuclei.

To confirm the terminal differentiation state of the human cells, expression of Myosin Heavy chain (*MYH2*, *3*, *8*), the sarcomeric proteins Tropomyosin1 (*TPM1*) and Actinin α3 (*ACTN*3) and creatine kinase (*CKM*) were analysed by RT-qPCR (Fig 3G–3J). PEG-Fibrin gels were not analysed due to the inability to extract sufficient mRNA. Human myotubes in Fibrin gel had an increased expression of *MYHC2*, *3*, *8* and *ACTN3* compared to Collagen (Fig 3G and 3H). As we are interested in biomaterials with potential clinical application, we did not formally evaluate Matrigel, since it is derived from Engelbreth-Holm-Swarm mouse sarcomas. However, Matrigel is widely used in the culture of myoblasts and to improve myotube formation, and so we did use Matrigel as a positive control. Matrigel was added to the Fibrin gel (10%: final concentration of 1mg/ml), which led to increased expression of *MYHC2*, *3*, *8*, *TPM1*, *ACTN*3 and *CKM* compared to Collagen 1 (Fig 3G–3J).

The dimensions of Collagen and Fibrin gels with embedded human myoblasts were also analysed at 0, 3 and 7 days of differentiation. Lengths of the 3D scaffolds were unchanged (data not shown). The molds were 2.5 mm wide, and so the 3D cultures began at this width, but Collagen gels with human myoblasts then became more compact (width of 1.48±0.02 mm) compared to Fibrin gels (2.30±0.08 mm) after the 2 day proliferation phase, when measured at Day 0 (Parts A and B of S1 Fig). Between day 0 and day 7 though, Fibrin gels further compacted during myogenic differentiation (Part B of S1 Fig). Addition of 10% Matrigel made Fibrin gels become more compact after 2 days of human myoblast proliferation, when measured at Day 0 (1.57±0.10 mm), with width then remaining unchanged through differentiation (Parts B and C of S1 Fig).

### Collagen I, Fibrin and PEG-Fibrinogen 3D scaffolds support limited differentiation of expanded primary murine satellite cells

**Fig 4:**
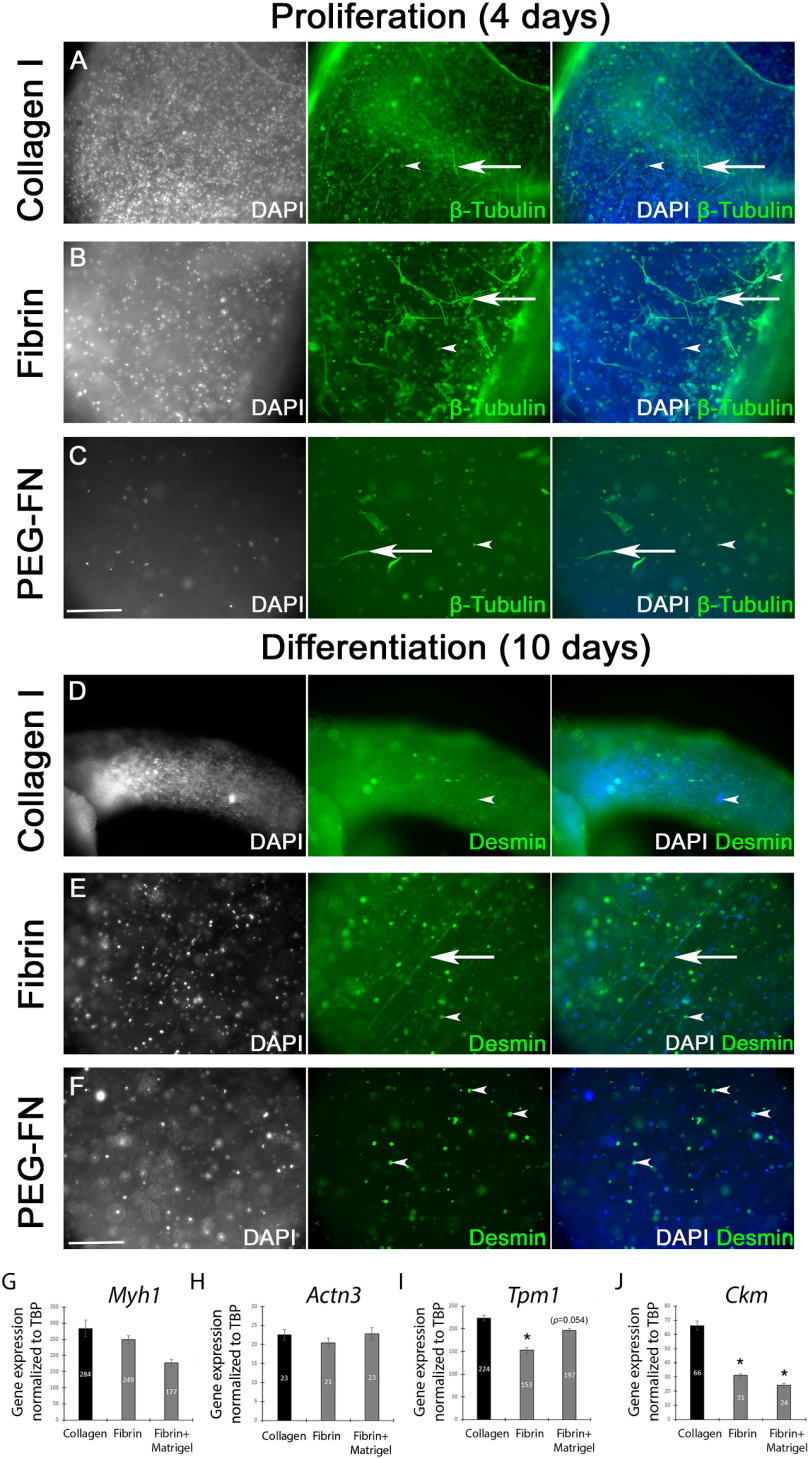
Limited myogenic differentiation of expanded primary murine satellite cells in 3D biomaterial scaffolds. *In vitro* expanded primary satellite cell-derived myoblasts were embedded in collagen I (500, 000 cells/100 μl: A and D), Fibrin (500, 000 cells/100 μl: B and E) or PEG-Fibrinogen (500, 000 cells/100 μl: C and F) and were cultured in proliferation medium for 4 days (A-C) and then switched to differentiation medium for 10 days (D-F). Cellular morphology of proliferating myoblasts (A-C) and myotubes (D-F) were visualized by immunolabelling for β-tubulin and Desmin respectively, and nuclei counterstained with DAPI. After 4 days in proliferation medium (A, B and C) most of the satellite cells had a rounded shape (arrow heads) and only few were elongated (arrows). Even after 10 days in differentiation medium, no myotubes were observed in collagen I (D) or PEG-Fibrinogen scaffolds (F) and only a few thin myotubes were present in Fibrin gel (arrow in E). Scale bar represents 100 μm. Representative images from 3 independent experiments. Expression of the sarcomeric proteins *myosin heavy chain (Myh1*) (G), tropomyosin 1 *(Tpm1*) (H) and actinin α3 *(Actn3*) (I) and creatine kinase *(Ckm)* (J) were analysed by RT-qPCR (G-J). Expression was normalized to the house keeping gene *Tbp*. Data are mean±SEM from satellite cells isolated from 3 mice where an asterisk denotes a significant difference (*p*<0.05) from Collagen gels using a paired two-tail Student's t-test.

We next analysed the behaviour of *ex vivo* expanded primary murine satellite cells, which are a more biologically pertinent model compared to immortalized C2C12. Satellite cells were isolated from the EDL muscle and expanded *ex vivo* on Matrigel for five days to obtain sufficient cells. Expanded primary satellite cell-derived myoblasts were embedded in collagen I (Fig 4A and 4D), Fibrin (Fig 4B and 4E) or PEG-Fibrinogen (Fig 4C and 4F), cultured in proliferation medium for 4 days (Fig 4A–4C) and then switched to differentiation medium for 10 days (Fig 4D–4F). Proliferating myoblasts were immunolabelled for β-Tubulin (Fig 4A–4C) and myotubes for Desmin, a muscle specific intermediate filament protein (Fig 4D–4F). After 4 days in proliferation medium (Fig 4A, 4B and 4C) most satellite cells were round, with only a few showing an elongated morphology. The inability of satellite cells to elongate could be an issue of cell-scaffold interaction and initial adhesion within the 3D environment. After ten days in differentiation medium, no myotubes were observed in collagen I (Fig 4D) and PEG-Fibrinogen scaffolds (Fig 4F) and only few thin myotubes were detected in Fibrin gels (arrow in Fig 4E).

To confirm terminal differentiation of expanded satellite cells embedded in 3D gels, expression of *Myh1*, *Actn3*, *Tpm1*, and *Ckm* was again analysed by RT-qPCR (Fig 4G–4J). PEG-Fibrin gels were not analysed due to the inability to extract enough mRNA for the analysis. Presence of all terminal differentiation markers was confirmed in all the biomaterials, but surprisingly compared to Collagen gels, satellite cells in Fibrin presented significantly less expression of *Tpm1* and *Ckm* (Fig 4I and 4J).

To determine if the lack of myogenic differentiation of *ex vivo* expanded primary murine satellite cells was due to the biomaterials, we added Matrigel (10%) into Fibrin gel as a positive control, since it is routinely used to enhance myotube formation. After only 2 days in differentiation medium, we observed robust spontaneous contraction (S1 Movie) in Fibrin/Matrigel hybrid gels that was not observed in Fibrin gels (S2 Movie). This confirmed that *ex vivo* expanded primary murine satellite cells were capable of robust differentiation and highlights the crucial function of ECM/growth factors supplied by Matrigel for satellite cell function in a 3D environment. Interestingly, though expanded satellite cells in Fibrin/Matrigel hybrid gels exhibited spontaneous contraction (S1 Movie), they did not show any difference in expression of sarcomeric genes compared to collagen 1 (Fig 4G–4I).

Dimensions of PEG-FN, Collagen and Fibrin gels were analysed during proliferation and differentiation of expanded satellite cells (S2 Fig). Diameter of the PEG-FN gels did not change during satellite cell proliferation but slightly increased during their differentiation (Part A of S2 Fig). Collagen gel width was unchanged under either satellite cell proliferation or differentiation conditions (Part B of S2 Fig). However, the width of Fibrin gels reduced during proliferation (from 2.55±0.05 mm to 1.92±0.02 mm) and even further during differentiation (1.55±0.03 mm after 2 days and 1.35±0.05 mm at day 3) (Part C of S2 Fig), suggesting a strong remodelling of Fibrin by satellite cells.

### Robust and functional myogenic differentiation of satellite cells delivered in their niche on a myofibre in 3D scaffolds

**Fig 5:**
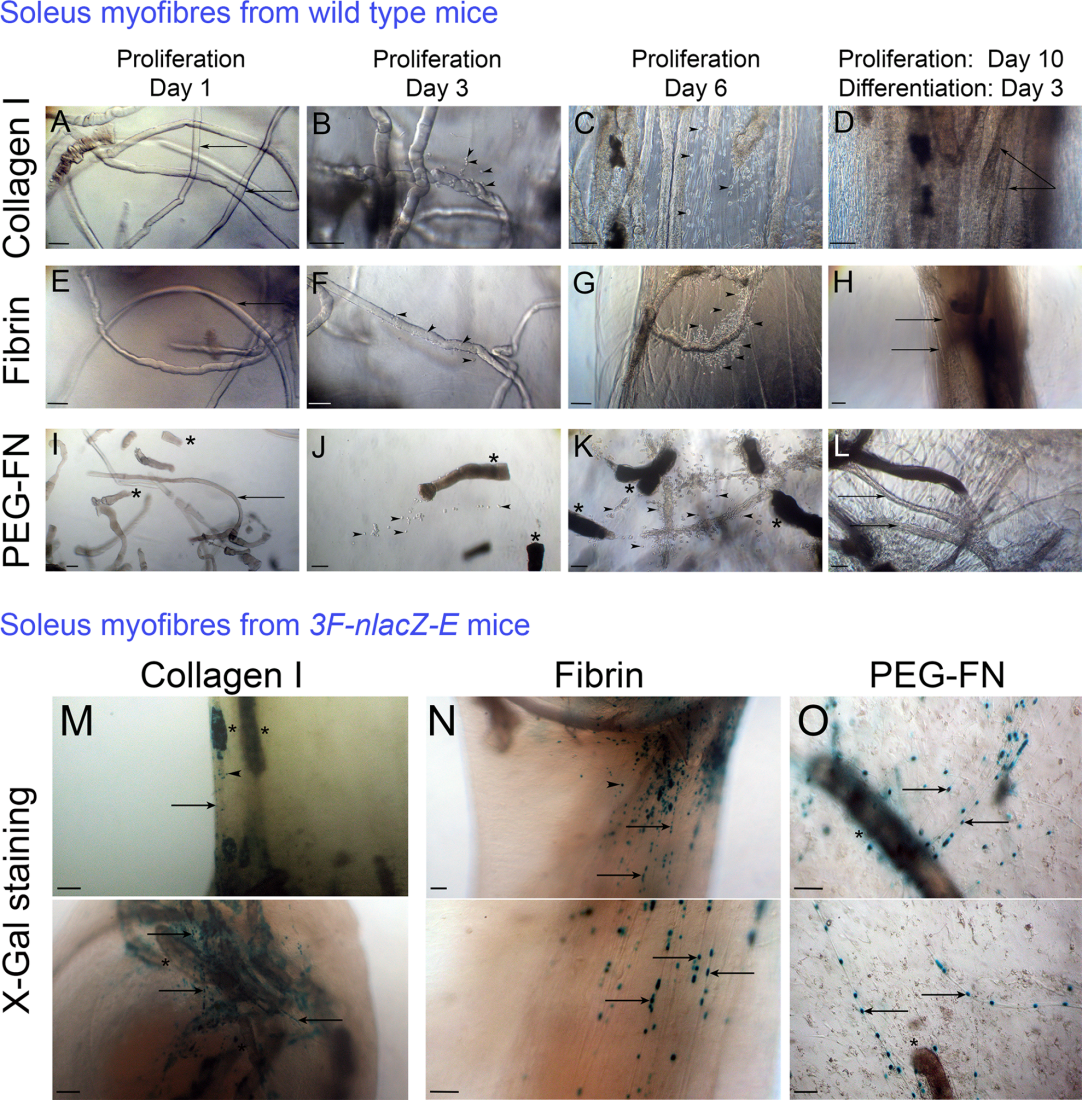
Satellite cells delivered in their niche on a myofibre make contractile myotubes in 3D scaffolds. Approximately 100 freshly isolated Soleus myofibres were embedded in 100 μl of collagen I (A-D), 100 μl of Fibrin (E-H) or 100 μl PEG-Fibrinogen (I-L) and were cultured in proliferation medium for 1 (A, E and I), 3 (B, F and J) or 6 days (C, G and K). After 10 days in proliferation medium, 3D scaffolds were switched to differentiation medium for 3 days (D, H and L). After 1 day (A, E and I), intact myofibres (arrows) were visible and some hypercontracted myofibres (asterisk) were observed in PEG-Fibrinogen (I). After 3 days (B, F and J), satellite cells (arrow heads) were present at the surface of myofibres and some had migrated into the biomaterial scaffold. After 6 days (C, G and K) satellite cells were still proliferating. After 10 days in proliferation medium and 3 days in differentiation medium (D, H and L) multinucleated, matured and functional myotubes (arrows) were observed. Representative images from repeats using myofibres from 3 mice. Scale bars represent approximately 100 μm. Freshly isolated Soleus myofibres from *3F-nlacZ-E* transgenic mice were embedded in collagen I (M), Fibrin (N) or PEG-Fibrinogen (O) gels. Biomaterial scaffolds were incubated in proliferation medium for 10 days, switched to differentiation medium for 3 days and then fixed and stained in X-gal solution to reveal β-galactosidase activity in myonuclei from the *3F-nlacZ-E* transgene. Differentiated X-Gal positive myonuclei were detected in myotubes (arrows). More X-Gal positive nuclei and parallel aligned myotubes were observed in Fibrin (N) compared to Collagen I (M). Representative images of 3 independent experiments using myofibres from 3 x *3F-nlacZ-E* mice.

*In vivo* transplantation of freshly isolated satellite cells while still in their niche on a muscle fibre, vastly improves their regenerative potential [6]. To test if the regenerative potential of satellite cells could be preserved in an *in vitro* 3D environment, we embedded freshly isolated myofibres in 3D scaffolds. 100 freshly isolated Soleus myofibres were embedded in 100 μl of collagen I (Fig 5A–5D), Fibrin (Fig 5E–5H) or PEG-Fibrinogen (Fig 5I–5L), the gels polymerised and then cultured in proliferation medium for 1 (Fig 5A, 5E and 5I), 3 (Fig 5B, 5F and 5J) or 6 (Fig 5C, 5G and 5K) days. After 10 days in proliferation medium, scaffolds were switched to differentiation medium for 3 days (Fig 5D, 5H and 5L).

After 1 day in proliferation medium, intact myofibres were present (Fig 5A, 5E and 5I), but more hypercontracted myofibres were observed in PEG-Fibrinogen (Fig 5I). In all biomaterial scaffolds, after 3 days in proliferation medium, activated satellite cells were observed migrating from their myofibre niche into the 3D matrix (Fig 5B, 5F and 5J). After 6 days, satellite cell-derived myoblasts were still proliferating, indicated by an increased number of cells present at the surface of the myofibres and inside the biomaterial gels (Fig 5C, 5G and 5K). After 10 days in proliferation medium followed by 3 days in differentiation medium, multinucleated myofibres were observed (Fig 5D, 5H and 5L). The static force exerted on the collagen I (Fig 5D) and Fibrin (Fig 5H) gels facilitated parallel alignment of myotubes. In absence of tension in the PEG-Fibrinogen scaffold, myotubes were randomly orientated (Fig 5L).

To evaluate myogenic differentiation of satellite cells delivered in their niche on a myofibre, we isolated Soleus myofibres from the *3F-nlacZ-E* mouse: transgenic for a construct with regulatory elements of the myosin light chain (MLC) 1/3F gene driving *nlacZ* reporter gene expression in myonuclei [72]. X-gal staining of satellite cells isolated from the *3F-nlacZ-E* mouse allows high resolution detection of their differentiated progeny, with β-galactosidase activity only in myonuclei. Freshly isolated Soleus myofibres from *3F-nlacZ-E* mice were embedded in collagen I (Fig 5M), Fibrin (Fig 5N) or PEG-Fibrinogen (Fig 5O) gels, incubated in proliferation medium for 10 days and then switched to differentiation medium for 3 days. Biomaterial gels were then fixed and stained in X-gal solution to detect transgenic β-galactosidase activity in myonuclei (Fig 5M–5O). More myonuclei were stained, and more parallel aligned myotubes were observed, in the Fibrin gel (Fig 5N) compared to collagen I (Fig 5M) or PEG-Fibrinogen (Fig 5O).

Importantly, myotubes formed from satellite cells delivered in their niche in all three 3D biomaterial gels showed spontaneous contraction, confirming their state of maturation and functionality (collagen I: S3 Movie; Fibrin: S4 Movie and PEG-Fibrinogen: S5 Movie). Movement and frequency of myotube contraction was higher in Fibrin scaffolds compared to collagen I scaffolds. Interestingly, multinucleated and contractile myotubes could be maintained for up to two months in 3D Fibrin gels. Thus, delivery of satellite cells in their niche on freshly isolated myofibres allows for robust proliferation and formation of multinucleated, mature and functional myotubes in a 3D environment with all three biomaterials tested.

## Discussion

In this study, we evaluated in parallel the ability of collagen I, Fibrin and PEG-Fibrinogen 3D scaffolds to support proliferation and myogenic differentiation of different types of muscle cells *in vitro*. The 3D culture system was originally developed for engineered heart tissue by embedding cardiomyocytes in Fibrin [68]. Here, we used this system for engineering skeletal muscle tissue by embedding myoblasts in collagen I and Fibrin gels. Polymerisation of Fibrin was stable, however with collagen I after polymerisation, a detachment from one of the flexible silicone posts was sometimes observed. We also tried to polymerise PEG-Fibrinogen between the two silicone posts using UV light. However, no polymerisation was observed probably due to an obstruction of the UV light by the silicone rack holding the posts.

This *in vitro* 3D culture system involves securing the biomaterial scaffold between two flexible silicone posts that mimic tendons, and so create predictable lines of tension [60]. Using this system, we observed an alignment of cells along the principal axis of force in collagen I and Fibrin gels. Such static force allows formation of parallel, aligned myotubes that are characteristic of native skeletal muscle and essential to efficiently generate force. Therefore, this simple 3D system represents a suitable *in vitro* model to analyse myogenesis, by allowing formation of aligned multinucleated and mature myotubes. Myofibre diameter and force production of *in vitro*-engineered skeletal muscle are known to be lower compared to native skeletal muscle and will be difficult to reach *in vitro* without more complex, multi-lineage culture to induce vascularisation and innervation [33, 51].

Static force during 3D culture of immortalized human C25Cl48 myoblasts produced myoblast and myotube alignment. However, compared to immortalized murine C2C12, only a moderate myotube formation was observed and no obvious difference was observed between collagen I and Fibrin gel. Human myoblasts were unable to contract spontaneously, and we did not try mechanical or electrical stimuli in this 3D system, that could improve myotube formation and maintenance. Alternatively, drugs can be added in the culture medium to stimulated myotube formation (e.g. HGF, FGF, IGF-1, DEX)[23] or muscle contraction (e.g. caffeine) [74]. Similarly, co-culture with fibroblasts can also improve myotube formation [75]. Finally, no other components were added routinely to the biomaterials that were tested, except Matrigel as a positive control, which contains the main component of the muscle stem cells niche (e.g. laminin) [35]. Other human myoblast clones may be able to differentiate better in this system though.

Using *ex vivo* expanded murine satellite cells with the 3D biomaterials, we observed only a moderate ability of the cells to fuse to form multinucleated myotubes. However, addition of growth factor reduced Matrigel to the Fibrin gel was able to enhance greatly the formation of contractile myotubes. Moreover, culture of satellite cells in their niche on freshly isolated myofibres in these 3D gels promoted the expansion of satellite cell-derived myoblasts and their robust differentiation and fusion into multinucleated, mature and contractile myofibres: showing greater regenerative capacity, in agreement with *in vivo* data [6, 10–12, 76]. This highlights the importance of biochemical and biophysical cues supplied by the native niche to maintain the regenerative potential of muscle stem cells [77]. Encouragingly then, primary murine satellite cells on a myofibre, compared to expanded satellite cells, behave in a similar way in our *in vitro* 3D culture system as when grafted *in vivo*.

Classic 2D culture of myoblasts is commonly used due to multiple advantages, such as no need for specific or complex equipment, fewer cells required, and fast, easy and reproducible quantification of gene expression by quantitative RT-qPCR and immunolabelling. However, in the absence of mechanical/electrical stimulation, critical for muscle cell viability [78], myotubes in 2D cell culture can only usually be maintained for few days as they start to spontaneously contract and soon detach from the culture surface. Using the 3D culture system however, with approximately 100 freshly isolated Soleus fibres (so only around 2500 satellite cells [79]), we obtained multinucleated and contractile myofibres with parallel alignment that generated force for a long period. Indeed, multinucleated and contractile myotubes were maintained for up to two months in 3D Fibrin gels. The spontaneous muscle contraction observed possibly plays a functional role in the integrity and maintenance of *in vitro* engineered muscle [78].

Apart from the influence on satellite cell function of remaining in their native niche, the isolated myofibres themselves could provide structural support and produce factors (e.g. IGF-1) [80] that could promote myotube formation. In addition, some residual fibroblasts may be present, which could also enhance differentiation [75, 81, 82]. This simple 3D system allows spontaneous contraction of myotubes without any external electrical stimulation or addition of molecules to induce muscle contraction. Therefore, this 3D *in vitro* culture system of myofibres could be a useful tool to analyse growth, maturation or muscle contraction in different contexts.

A video optical recording system developed for cardiomyocytes [68] will be a valuable tool to automatically evaluate the contractile activity of myotubes. The system could also be used to test drugs that could affect muscle mass (atrophy/hypertrophy) over a longer period. *In vitro*, culture of neonatal rat myogenic cells embedded in Fibrin can generate a functional muscle with resident satellite cells that allow muscle regeneration *in vitro* [51]. It will also be interesting to check if our system also allows the formation of a new satellite cells niche and supports the self-renewal of satellite cells. However, in our hands, sufficient optical resolution could not be obtained to investigate this.

The 3D PEG-Fibrinogen scaffold allows myotube formation of murine myoblasts but myotubes were not aligned and smaller compared with collagen I and Fibrin gels. Generally, we observed less remodelling of the PEG-Fibrinogen gel and more rounded cells inside the matrix. These results suggest that myoblasts have a reduced adhesion with the biosynthetic matrix and are less able to remodel the matrix. However, at the same time, Fibrin gels require stabilisation with Aprotinin to prevent degradation by cells, and the higher stability of the PEG-Fibrinogen could provide useful in certain scenarios. PEG-Fibrinogen was not polymerised between the two silicone posts, but in a 96 well plate. The resulting shape of PEG-Fibrinogen gels most likely influenced the diffusion of nutrients and oxygen. Moreover, the absence of static force applied to the scaffold likely explains lack of myotube alignment and their reduced size. *In vivo*, transplantation of mesoangioblasts in PEG-Fibrinogen generated a complete and functional (vascularised, innervated and contractile) muscle [32]. Using freshly isolated murine muscle fibres embedded in PEG-Fibrinogen we also observed a robust formation of multinucleated and contractile myotubes. Our data supports the potential of the PEG-Fibrinogen scaffold to sustain myotube formation of murine satellite cells *in vitro* when delivered on their freshly isolated muscle fibres.

In conclusion, we show that biomaterials collagen I, Fibrin and PEG-Fibrinogen supported myoblast proliferation and differentiation. However, our study revealed that subtle differences exist between the way the same cell type interacts with different biomaterials, or how different cell types interact with the same material. Additionally, we highlight how important the cell source is: a hundred freshly transplanted soleus fibres with ~2500 satellite cells [79] greatly outperformed directly embedding 500 000 expanded satellite cell-derived myoblasts.

## Supporting information

S1 Fig Immortalized human myoblasts remodel 3D biomaterial scaffolds. Immortalized human C25Cl48 myoblasts were embedded in Collagen (A), Fibrin (B) or Fibrin with 10% Matrigel (final concentration of 1mg/ml) (C), cultured in proliferation medium for 2 days and then switched to differentiation medium for 7 days. The mold width, so initial gel width, was 2.5 mm (red line). Width of the 3D scaffolds was measured at three different time points, after the 2 days of proliferation (day 0), when cells were switched to differentiation medium and then after 3 and 7 days of differentiation. At 2 days of proliferation (day 0), Collagen and Fibrin/Matrigel hybrid were more compact (reduced width) compared to Fibrin (B). During the differentiation process a moderate reduction in width was observed in Fibrin gels. Data are mean±SEM from 3 independent gels where an asterisk denotes a significant difference (*p*<0.05) from D0 using an unpaired two-tail Student's t-test.
(TIF)

S2 Fig 3D biomaterial scaffolds remodelling with expanded primary murine satellite cells. *In vitro* expanded primary murine satellite cells were embedded in PEG-FN (A), Collagen I (B) or Fibrin (C) and cultured in proliferation medium for 4 days and then switched to differentiation medium. The dimensions of PEG-FN, Collagen and Fibrin gels was measured at several time points during proliferation and differentiation. The well diameter and mold width, so initial gel width, are indicated by a red line. The diameter of the PEG-FN gels did not change during satellite cell proliferation and slightly increased during their differentiation (A). Collagen gel width did not change during either satellite cell proliferation or differentiation (B). Fibrin gel width reduced during satellite cell proliferation and further during their differentiation (C). Data are mean±SEM from satellite cells isolated from 3 mice, where an asterisk denotes a significant difference (*p*<0.05) from day 0 of proliferation using a paired two-tail Student's t-test. Scale bars represent approximately 1 mm.
(TIFF)

S1 Movie In vitro expanded satellite cell-derived myoblasts in hybrid Fibrin/Matrigel 3D scaffold. *In vitro* expanded primary murine satellite cells were embedded in Fibrin with 10% Matrigel, cultured in proliferation medium for 4 days and then switched to differentiation medium for 2 days. After 2 days of differentiation, robust spontaneous contraction was observed in the 3D scaffold. Representative data from 3 independent gels containing *in vitro* expanded murine satellite cells from 3 mice.
(MP4)

S2 Movie *In vitro* expanded satellite cell-derived myoblasts in Fibrin 3D scaffold. *In vitro* expanded primary murine satellite cells were embedded in Fibrin, cultured in proliferation medium for 4 days and then switched to differentiation medium for 2 days. After 2 days of differentiation no spontaneous contraction was observed. Representative data from 3 independent gels containing *in vitro* expanded murine satellite cells from 3 different mice.
(MP4)

S3 Movie Formation of contractile myotubes from murine satellite cells delivered in their niche on a myofibre in 3D collagen I gels. Freshly isolated Soleus myofibres were embedded in a collagen I gel, cultured in proliferation medium for 10 days and then switched to differentiation medium for 3 days. Some hypercontracted myofibres (asterisks) were observed. Functional myotubes exhibiting spontaneous contractions were present (arrows). Representative data from 3 independent gels using myofibres from 3 mice.
(MP4)

S4 Movie Formation of contractile myotubes from murine satellite cells delivered in their niche on a myofibre in 3D Fibrin scaffold. Freshly isolated Soleus myofibres were embedded in fibrin gel, cultured in proliferation medium for 10 days and then switched to differentiation medium for 3 days. Large functional contractile myotubes (arrows) were observed, producing spontaneous force strong enough to move the flexible silicone posts. Representative data from 3 independent gels using myofibres from 3 mice.
(MP4)

S5 Movie Formation of contractile myotubes from murine satellite cells delivered in their niche on a myofibre in 3D PEG-Fibrinogen scaffold. Freshly isolated Soleus myofibres were embedded in PEG-Fibrinogen, cultured in proliferation medium for 10 days and then switched to differentiation medium for 3 days. Several functional contractile myotubes (arrow heads) were observed but without alignment or specific orientation. Representative data from 3 independent gels using myofibres from 3 mice.
(MP4)
