# Supplementary figures and images for: Satellite cells delivered in their niche efficiently generate functional myotubes in three-dimensional cell culture
Source: PLoS One. 2018 Sep 17;13(9):e0202574. doi: 10.1371/journal.pone.0202574 (PMC6141091; doi:10.1371/journal.pone.0202574)

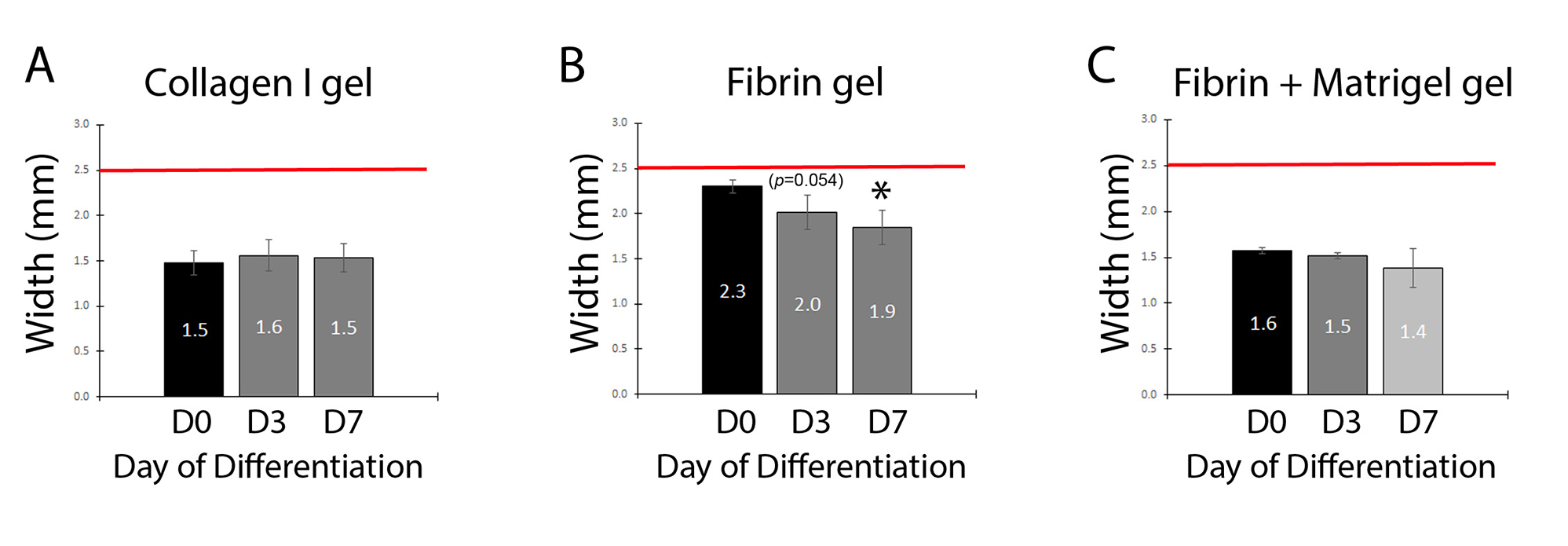

Supplement: S1 Fig — Immortalized human C25Cl48 myoblasts were embedded in Collagen (A), Fibrin (B) or Fibrin with 10% Matrigel (final concentration of 1mg/ml) (C), cultured in proliferation medium for 2 days and then switched to differentiation medium for 7 days. The mold width, so initial gel width, was 2.5 mm (red line). Width of the 3D scaffolds was measured at three different time points, after the 2 days of proliferation (day 0), when cells were switched to differentiation medium and then after 3 and 7 days of differentiation. At 2 days of proliferation (day 0), Collagen and Fibrin/Matrigel hybrid were more compact (reduced width) compared to Fibrin (B). During the differentiation process a moderate reduction in width was observed in Fibrin gels. Data are mean±SEM from 3 independent gels where an asterisk denotes a significant difference (p<0.05) from D0 using an unpaired two-tail Student's t-test. (TIF) [file pone.0202574.s001.tif]

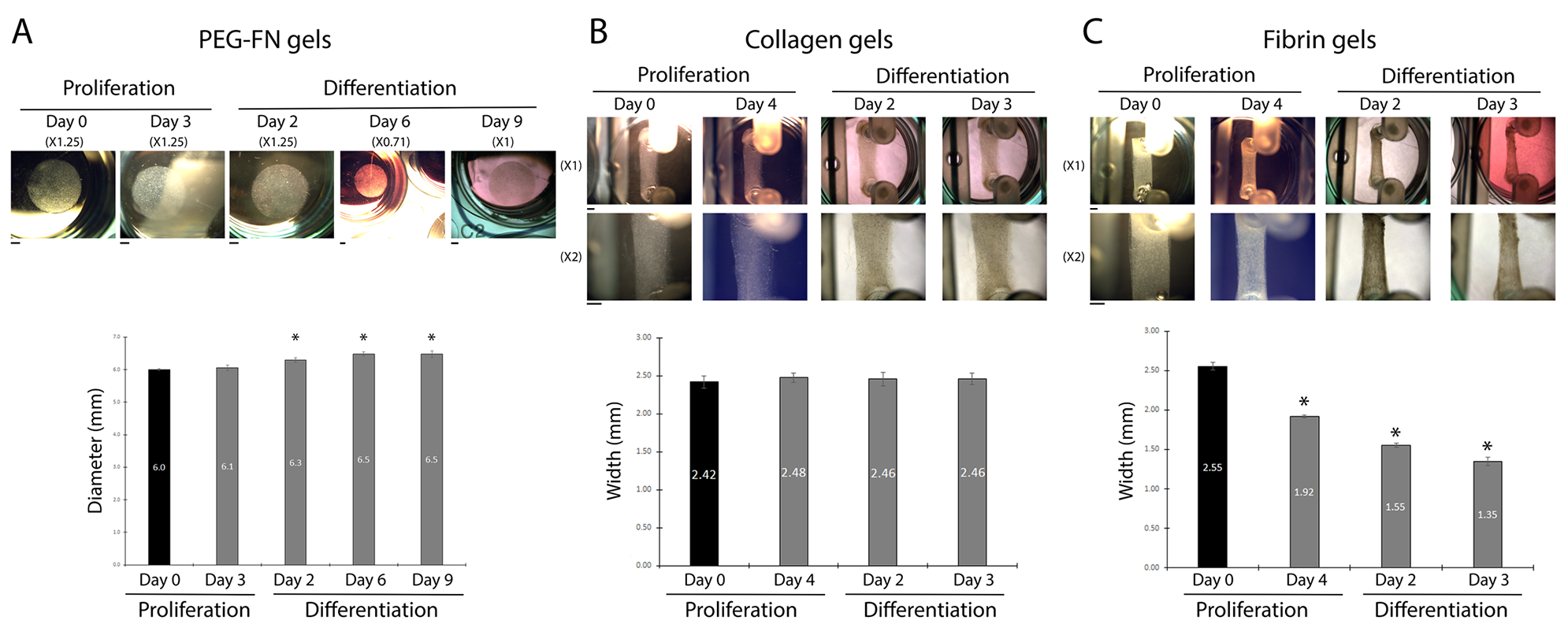

Supplement: S2 Fig — In vitro expanded primary murine satellite cells were embedded in PEG-FN (A), Collagen I (B) or Fibrin (C) and cultured in proliferation medium for 4 days and then switched to differentiation medium. The dimensions of PEG-FN, Collagen and Fibrin gels was measured at several time points during proliferation and differentiation. The well diameter and mold width, so initial gel width, are indicated by a red line. The diameter of the PEG-FN gels did not change during satellite cell proliferation and slightly increased during their differentiation (A). Collagen gel width did not change during either satellite cell proliferation or differentiation (B). Fibrin gel width reduced during satellite cell proliferation and further during their differentiation (C). Data are mean±SEM from satellite cells isolated from 3 mice, where an asterisk denotes a significant difference (p<0.05) from day 0 of proliferation using a paired two-tail Student's t-test. Scale bars represent approximately 1 mm. (TIFF) [file pone.0202574.s002.tiff]
